# Effects of tree mycorrhizal dominance on soil microbial community structure and microbial nutrient limitation

**DOI:** 10.3389/fmicb.2025.1698121

**Published:** 2026-01-08

**Authors:** Yajie Xu, Longfei Hao, Yongjie Yue, Runhong Gao, Lingze Zhang, Kai Zhao, Zhenghui Zhao

**Affiliations:** 1 College of Forestry, Inner Mongolia Agricultural University, Hohhot, China; 2 College of Desert Control Science and Engineering, Inner Mongolia Agricultural University, Hohhot, China

**Keywords:** arbuscular mycorrhizal fungi, ectomycorrhizal fungi, mixed-mycorrhizal strategy, microbial nutrient limitation, soil extracellular enzyme activity, microbial community structure

## Abstract

**Introduction:**

Mycorrhizal fungi play a central role in nutrient cycling in forest ecosystems. The functional differences between arbuscular mycorrhizal (AM) and ectomycorrhizal (EcM) tree species significantly affect soil microbial community structure and patterns of microbial nutrient limitation, with substantial implications for ecosystem stability and biogeochemical cycling under global changes. However, the regulatory mechanisms of different dominant mycorrhizal tree species and their mixed mycorrhizal configurations on microbial nutrient limitation remain unclear.

**Methods:**

This study investigated a typical AM tree species (*Ulmus pumila*) and an EcM tree species (*Pinus sylvestris var. mongolica*) in the southern Horqin Sandy Land. We compared rhizosphere soil nutrient status, extracellular enzyme activities, and microbial community structure among pure *U. pumila* stands, pure *P. sylvestris* var. mongolica stands, and *U. pumila*–*P. sylvestris* mixed stands.

**Results:**

Results showed that AM pure forests exhibited extremely high C-acquiring enzyme activities but reduced soil nutrient content and microbial biomass, maintaining higher bacterial diversity. By contrast, the activities of N- and P-acquiring enzymes, soil nutrient contents, and fungal diversity in the EcM pure stands were significantly higher than those in the AM pure stands (*p* < 0.05). Mixed forests improved soil nutrient status through complementary mixed-mycorrhizal strategies, promoted microbial biomass accumulation, and modulated extracellular enzyme activities, thereby significantly alleviating microbial nutrient limitations. Moreover, their microbial networks exhibited greater complexity and stability than pure stands. Structural equation modeling further revealed that tree mycorrhizal dominance and microbial biomass were the primary factors alleviating microbial nutrient limitation: microbial biomass and mycorrhizal dominance showed significant negative effects on vector length (path coefficients −0.71 and 0.33, *p* < 0.01), whereas mycorrhizal dominance exerted a highly significant positive effect on vector angle (path coefficient 0.70, *p* < 0.001).

**Discussion:**

In conclusion, mixed mycorrhizal strategies alleviate microbial nutrient limitations by enhancing soil nutrient status, microbial community structure, and extracellular enzyme activities, providing theoretical support for ecosystem restoration and sustainable development in arid regions.

## 1 Introduction

Almost all tree species are associated with either arbuscular mycorrhizal (AM) fungi or ectomycorrhizal (EcM) fungi (Phillips et al., 2013). These fungi form symbiotic relationships with trees, exchanging carbon (C) as a nutrient source and protecting trees from environmental stresses (Brundrett and Tedersoo, 2018). Tree species associated with AM and EcM fungi differ in litter quality, root and hyphal traits, and nutrient uptake efficiency, which strongly influence biogeochemical processes (C and nutrient cycling) in ecosystems (Zheng et al., 2023; Bahram et al., 2020; Tedersoo and Bahram, 2019). In general, EcM-associated trees are characterized by lower-quality litter, slower decomposition, higher forest floor C storage, and elevated soil C/N ratios compared with AM-associated trees (Craig et al., 2018; Jo et al., 2019). According to the mycorrhiza-associated nutrient economy framework, AM trees are linked to an inorganic nutrient economy with rapid nutrient cycling, whereas EcM trees are linked to an organic nutrient economy with slow turnover of plant-derived C and strong root–microbe interactions (Yu et al., 2023). Differences in mycorrhizal type are also consistent with pronounced shifts in soil microbial composition, activity, and function, which can alter soil organic carbon (SOC) and nutrient turnover (Heděnec et al., 2020; Tang et al., 2024a). Currently, most studies remain focused on the ecological effects of single dominant mycorrhizal types, whereas systematic understanding of the role of AM–EcM mixed forests in regulating soil biogeochemical processes and microbial nutrient limitations is still lacking. In recent years, a gradual shift in forest management from monoculture plantations to mixed-species stands has emerged as an important strategy to accelerate the restoration of degraded forests and enhance ecosystem services (Deng et al., 2020). Studies suggest that litter from AM-associated trees may accelerate the decomposition of needle litter by adding labile nutrients and stimulating microbial metabolism, thereby improving soil ecosystem stability (Polyakova and Billor, 2007; Zhang et al., 2020). Tang et al. (2024b) further demonstrated that introducing native broadleaf trees into coniferous plantations promotes biogeochemical cycling in Masson pine forests and strengthens ecosystem functions. Thus, combining different mycorrhiza-associated tree species may facilitate efficient resource use, enhancing forest ecosystem stability.

Soil microbes regulate C and nutrient transformations and cycling through the secretion of extracellular enzymes, thereby obtaining energy and nutrients to meet their metabolic requirements. Moreover, shifts in microbial extracellular enzyme activities significantly affect microbial metabolic functions, nutrient demands, and stoichiometric balance (Zhou et al., 2020; Cui et al., 2021). Previous studies have shown that *β*-1,4-glucosidase (BG), *β*-1,4-*N*-acetylglucosaminidase (NAG), leucine aminopeptidase (LAP), and acid or alkaline phosphatase (AP) serve as indicators of microbial C, N, and P demands, respectively (Sinsabaugh et al., 2009; Liu et al., 2023; Feng et al., 2019). Ecoenzymatic stoichiometry connects microbial nutrient acquisition strategies with soil nutrient availability, reflecting the supply–demand balance of nutrients for soils and microbes. Over the past decades, enzyme activity and ecoenzymatic stoichiometry, including vector analysis, have been widely applied to assess microbial metabolic activities and resource limitations in ecosystems (Wang et al., 2023). For example, Moorhead et al. (2013) proposed calculating vector length and angle based on (BG/[BG + ALP]) and (BG/[BG + NAG + LAP]) to quantify relative C versus nutrient acquisition (vector length) and P versus N acquisition (vector angle). Mycorrhizal dominance of tree species (i.e., pure stands dominated by one mycorrhizal type vs. mixed stands composed of AM- and EcM-associated trees) can lead to distinct soil nutrient regimes and microbial metabolic strategies, triggering specific resource acquisition strategies (Luo et al., 2023). Therefore, exploring the relationships between mycorrhizal dominance and microbially mediated soil processes is critical for optimizing stand structure, enhancing forest ecosystem functions, and responding to global environmental changes.

Semi-arid regions account for 37% of China’s land area and play a critical role in national ecological security and ecological civilization. Aohan County, Chifeng City, Inner Mongolia, is a typical semi-arid region known as China’s “first county of afforestation,” with a forest cover of 44.07%. However, local forests face widespread soil fertility decline and reduced ecosystem functions due to prolonged drought, extreme climate, nutrient-poor soils, and long-term monoculture plantations. In light of the ongoing escalation of global climate change and regional ecological pressure, the stability and adaptability of artificial forests must be urgently improved through scientific means. Thus, a thorough understanding of mycorrhizal dominance and microbially mediated nutrient cycling is essential for restoring soil fertility in degraded plantations, optimizing stand composition, and improving ecological functions and sustainable forest management. The present study focused on the typical AM-associated tree species (*Ulmus pumila*) and EcM-associated tree species (*Pinus sylvestris* var. *mongolica*), as well as their mixed stands, at the southern edge of the Horqin Sandy Land. We analyzed the rhizosphere soil microbial community structure and nutrient limitation patterns. We proposed the following hypotheses: (1) compared with EcM-associated trees, soils under AM-associated trees exhibit stronger microbial C limitation and greater bacterial dominance; and (2) resource complementarity strategies are present in AM + EcM mixed forests, which can improve the overall resource-use efficiency of the stands and mitigate microbial nutrient limitations.

## 2 Materials and methods

### 2.1 Overview of the study area

**Figure 1:**
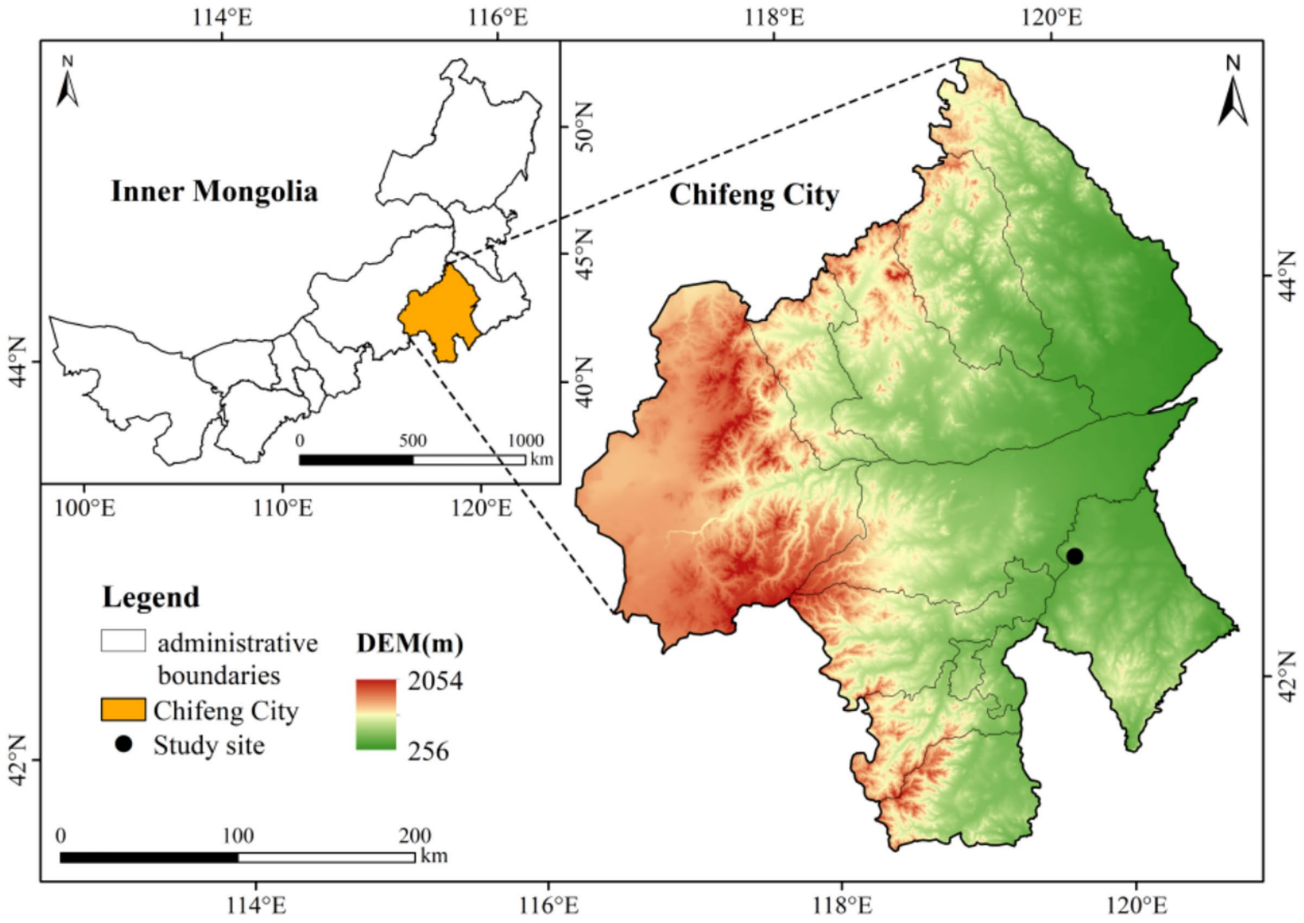
Location of the study area.

The study area is located in Aohan Banner, Chifeng City, Inner Mongolia Autonomous Region, at the southern margin of the Horqin Sandy Land (E 119°30′–120°53′, N 41°42′–43°02′). The region extends 176 km from north to south and 122 km from east to west, with a total land area of 829 km^2^. The climate is mainly continental monsoon type, with a large temperature difference between day and night. Spring is dry and windy with rapid warming; summer is hot with concentrated rainfall; autumn is marked by a sharp temperature drop; and winter is cold. The mean annual precipitation is approximately 351.8 mm, with a mean annual temperature of 8 °C. The extreme minimum and maximum temperatures are −31 °C and 39.7 °C, respectively, with a frost-free period of 130–150 days. The accumulated annual temperature ranges from 2,600 °C to 3,200 °C. The dominant soil types are brown soil, chestnut soil, and aeolian sandy soil, among which chestnut soil and sandy soil account for 52.6% of the total land area. The region is mainly covered by plantations, with *P. sylvestris* var. *mongolica*, *U. pumila*, and *Populus* spp. as the dominant species. *Prunus sibirica* and *Pinus tabuliformis* are also present (Figure 1).

### 2.2 Site selection and sample collection

We selected three forest stand types planted in 2008 at the Shuangjing Forest Farm in Aohan Banner, Inner Mongolia: *P. sylvestris* var. *mongolica* pure stands (EcM-associated tree species), *U. pumila* pure stands (AM-associated tree species), and mixed stands in which the two species were planted at a 1:1 ratio. To ensure comparability, we established plots under similar site conditions (elevation, slope, and aspect). For each stand type, three replicate plots (50 m × 50 m) were established, resulting in nine plots in total, with an inter-plot distance of approximately 1,000 m. Soil samples were collected in late September 2024, ensuring that no rainfall had occurred for at least seven consecutive days prior to sampling. Using a tree-by-tree inventory, we selected 15 standard trees from each pure stand plot. In the mixed stand, 15 *P. sylvestris* var. *mongolica* and 15 *U. pumila* individuals were selected as standard trees in each plot. We took the standard trees as the center and collected rhizosphere soil samples within a horizontal radius of 1 m and a vertical soil layer of 0–20 cm by the shaking method. This soil layer represents the zone with the highest fine-root density and strongest rhizosphere effects (Cleveland and Liptzin, 2007), and it also exhibits the greatest extracellular enzyme activity and microbial activity, making it the most sensitive to microbial resource limitation (Saiya-Cork et al., 2002). Subsequently, the soil samples obtained from every 5 standard trees within the same standard plot were evenly mixed to obtain 1 soil sample. For each standard plot, 15 standard trees of the same tree species were mixed to form 3 soil samples. A total of 9 soil samples were obtained from each treatment. Soil samples from *P. sylvestris* var. *mongolica* pure stands, *U. pumila* pure stands, and from *P. sylvestris* var. *mongolica* and *U. pumila* in the mixed stands were labeled Ps.P, Up.P, Ps.M, and Up.M, respectively. Samples were sieved (2 mm) to remove roots and stones and then divided into three subsamples: one stored at 4 °C for microbial biomass, enzyme activity, and available nutrient analyses; one frozen at −80 °C for microbial community analysis; and one air-dried for soil total nutrients and organic matter determination.

### 2.3 Soil nutrient determination

The desktop electrode pH meter (Sartorius Basic pH Meter PB-10) was used to measure the pH of the soil at a ratio of 1:25 (w/v) with 0.01 M CaCl_2_ solution. Total carbon (TC) and total nitrogen (TN) were determined with a Vario EL elemental analyzer (Elementar, Hanau, Germany) (Zeng et al., 2010), whereas total phosphorus (TP) was measured using the molybdenum–antimony anti-spectrophotometric method (Tighe et al., 2018). SOC, available nitrogen (AN), and available phosphorus (AP) were measured by the potassium dichromate oxidation method, alkaline hydrolysis diffusion, and Olsen method, respectively (Mebius, 1960; Cornfield, 1960; Page et al., 1982).

### 2.4 Measurement of microbial biomass

Microbial biomass carbon (MBC), microbial biomass nitrogen (MBN), and microbial biomass phosphorus (MBP) were determined using the chloroform fumigation–extraction method (Shu et al., 2023). The C and N concentrations in K_2_SO_4_extracts from fumigated and non-fumigated samples were analyzed with a total organic carbon analyzer equipped with an N unit (TOC-V and TNM-1, Shimadzu, Japan). The P concentration in NaHCO_3_ extracts was determined with a continuous flow analyzer (BRAN+LUEBBE AA3, Germany). Conversion factors of kEC = 0.45, kEN = 0.54, and kEP = 0.40 were applied for MBC, MBN, and MBP, respectively (Wu et al., 1990; Brookes et al., 1985).

### 2.5 Determination of soil enzyme activity and quantification of microbial nutrient limitation

We selected four extracellular enzymes that represent the cycling of C, N, and P substances: BG, NAG, LAP, and ALP. Among them, BG is a C cycle-related enzyme, NAG and LAP are N cycle-related enzymes, and ALP is a P cycle-related enzyme. Enzyme assays followed the method of Saiya-Cork et al. (2002). A vector analysis was used to quantify the microbial metabolic limitation based on untransformed enzymatic activities (Xu et al., 2022). The length of the vector represents the microbial carbon limitation, and the microbial C limitation increases with the increase in vector length. An angle of the vector lower than 45° indicates microbial N limitation, whereas an angle of the vector higher than 45° indicates microbial P limitation. The microbial N limitation decreases with the increase in vector angle, and the microbial phosphorus limitation increases with the increase in vector angle. The vector length and angle can be calculated as follows (Moorhead et al., 2013):


$$\text{Vector length}=\sqrt{{\left(\ln(\mathrm{BG})/\ln(\mathrm{NAG}+\mathrm{LAP})\right)}^{2}+{\left(\ln(\mathrm{BG})/\ln(\mathrm{AP})\right)}^{2}}$$

$$\text{Vector length}=\text{DEGREES}\{\text{ATAN}2[\begin{array}{l} (\ln(\mathrm{BG})/\ln(\mathrm{AP})),\\ \left(\ln(\mathrm{BG})/\ln(\mathrm{NAG}+\mathrm{LAP})\right) \end{array}]\}$$

### 2.6 Determination of microbial community composition

According to the manufacturer’s instructions, the microbial community gene DNA was extracted from the soil samples using HiPure Soil DNA kits. The ITS2 region of ITS and the V3 + V4 region of 16S rDNA were amplified using specific primers with barcodes. The primer sequences were as follows: ITS3_KYO2: GATGAAGAACGYAGYRAA; ITS4: TCCTCCGCTTATTGATATGC and 341F: CCTACGGGNGGCWGCAG; and 806R: GGACTACHVGGGTATCTAAT. The amplified products (i.e., amplicons) were ligated with sequencing adapters, and a sequencing library was constructed. Sequencing was performed on the Illumina platform. The operational taxonomic units (OTUs) with 97% similarity were clustered using the UPARSE method (Edgar, 2013), and chimeric sequences were identified and removed. The OTUs obtained were checked using the MaarjAM database^1^ (Öpik et al., 2010), and unclassified OTUs were manually checked and filtered in the NCBI database (Jiang et al., 2018). The sequencing depth of the fungal ITS data in this study ranged from 67,329 to 78,875 valid sequences per sample, with an average of 73,428 sequences per sample; the sequencing depth of the bacterial 16S data ranged from 114,221 to 130,978 valid sequences per sample, with an average of 121,412 sequences per sample. These sequence data also served as the basis for the subsequent construction of microbial co-occurrence networks and the calculation of network topological properties. To eliminate the influence of sequencing depth differences on the *α* diversity analysis, the ITS and 16S data sets were, respectively, subjected to sparsification to achieve the minimum sequencing depth that could retain all samples in each dataset. The sparsified OTU table was used to calculate the Observed richness, Chao1, Shannon–Wiener, and Simpson indices.

### 2.7 Statistical analysis

Data analyses were performed using DPS (version 9.01), and data visualization was conducted in Origin (version 9.4). One-way analysis of variance (ANOVA) followed by Fisher’s least significant difference (LSD) test at a 5% significance level was used to evaluate rhizosphere soil physicochemical properties, extracellular enzyme activities, microbial biomass, and fungal and bacterial diversity. Principal component analysis (PCA) of soil extracellular enzyme activities was conducted using Origin (version 9.4). OTUs with a mean relative abundance <0.01% and occurring in less than 50% of the total samples were filtered out in R (version 4.2.1) Spearman’s rank correlations among OTUs were calculated using the “Hmisc*”* package, and only strong (|*r*| > 0.9) and significant (*p* < 0.05) associations were retained for constructing the co-occurrence network. Undirected weighted microbial co-occurrence networks were then constructed using the “igraph” and “ggClusterNet” packages, and network topological properties (including node number, edge number, average degree, average path length, clustering coefficient, and modularity) were calculated. The ecological interpretations of these topological metrics are as follows: the numbers of nodes and edges reflect the overall network size and the richness of potential interactions; the average degree represents the overall connectivity among taxa; the average path length indicates the compactness of the network and the potential efficiency of interactions; the clustering coefficient describes local connectivity and the aggregation of taxa; and modularity reflects the extent to which the network is organized into ecological niches or functional guilds (Ma et al., 2020). Network visualization was performed using Gephi v0.9.2 (Ding et al., 2025). The “mgcv*”* package in R (version 4.2.1) was used to fit generalized additive models (GAMs) to examine the relationships between microbial C and N limitations and soil pH, soil nutrients, and microbial biomass. Partial least squares path modeling (PLS-PM) was performed using the “plspm” package in R (version 4.2.1) to assess pathways influencing microbial resource limitation, including mycorrhizal dominance (i.e., rhizosphere soils of *Pinus sylvestris* var. mongolica monoculture, *Ulmus pumila* monoculture, and both species in the mixed stand), pH, soil nutrients (i.e., total carbon, total nitrogen, total phosphorus, organic carbon, available nitrogen, and available phosphorus), fungal diversity (i.e., fungal observed richness and Chao1 index), bacterial diversity (i.e., bacterial observed richness and Chao1 index), and microbial biomass (i.e., microbial biomass C, N, and P). To ensure model robustness, variables with loading values below 0.6—including the fungal Shannon–Wiener index, fungal Simpson index, bacterial Shannon–Wiener index, and bacterial Simpson index—were excluded (Liu et al., 2024).

## 3 Results

### 3.1 Influence of tree mycorrhizal dominance on soil chemical properties

The dominance of tree mycorrhizae had a significant impact on the chemical properties of the rhizosphere soil (*p* < 0.05, Table 1). TC, TN, TP, SOC, AN, and AP all reached their lowest values at Up.P, suggesting significant differences compared with other treatments (*p* < 0.05). By contrast, Up.M showed the highest TC (9.85 g kg^−1^), TN (0.58 g kg^−1^), SOC (4.05 g kg^−1^), and AN (114.91 mg g^−1^), indicating that the mixed forest significantly increased the organic carbon input and nitrogen accumulation levels in the AM-related rhizosphere soil, thereby promoting soil fertility. AP reached the highest value at Ps.P (2.73 mg g^−1^), with significant differences observed across each treatment (*p* < 0.05), suggesting that the rhizosphere soil of exogenous mycorrhizal tree species have certain advantages in obtaining and maintaining P. The mixed forest effectively improved the carbon and nitrogen nutrient conditions of the rhizosphere soil through the combination of AM and EcM tree species, and it exerted a certain regulatory effect on soil pH, facilitating the availability of soil nutrients and microbial functions.

**Table 1:** Soil physicochemical properties under different dominant mycorrhizal forests.

Treatment	pH	TC (g kg^−1^)	TN (g kg^−1^)	TP (g kg^−1^)	SOC (g kg^−1^)	AN (mg g^−1^)	AP (mg g^−1^)
Ps.P	8.11 ± 0.12 b	7.75 ± 0.17 b	0.45 ± 0.01 b	0.14 ± 0.01 ab	3.29 ± 0.14 c	100.87 ± 1.18 b	2.73 ± 0.10 a
Up.P	8.35 ± 0.16 a	3.80 ± 0.13 d	0.25 ± 0.01 d	0.09 ± 0.01 c	2.19 ± 0.11 d	54.54 ± 0.82 d	1.35 ± 0.08 c
Ps.M	8.17 ± 0.11 b	7.34 ± 0.13 c	0.43 ± 0.01 c	0.13 ± 0.01 b	3.77 ± 0.12 b	89.59 ± 1.08 c	2.38 ± 0.10 b
Up.M	7.76 ± 0.05 c	9.85 ± 0.18 a	0.58 ± 0.01 a	0.14 ± 0.01 a	4.05 ± 0.23 a	114.91 ± 1.61 a	2.32 ± 0.10 b

Ps.P, *Pinus sylvestris* pure stand; Up.P, *Ulmus pumila* pure stand; Ps.M, *Pinus sylvestris* in mixed stand; Up.M, *Ulmus pumila* in mixed stand. TC, total carbon; TN, total nitrogen; TP, total phosphorus; SOC, soil organic carbon; AN, available nitrogen; and AP, available phosphorus. Significant differences (*p* < 0.05) between treatments are indicated by different lowercase letters. Values are means ± SE (*n* = 9).

### 3.2 Impacts of tree mycorrhizal dominance on soil extracellular enzyme activity and microbial resource limitation

**Figure 2:**
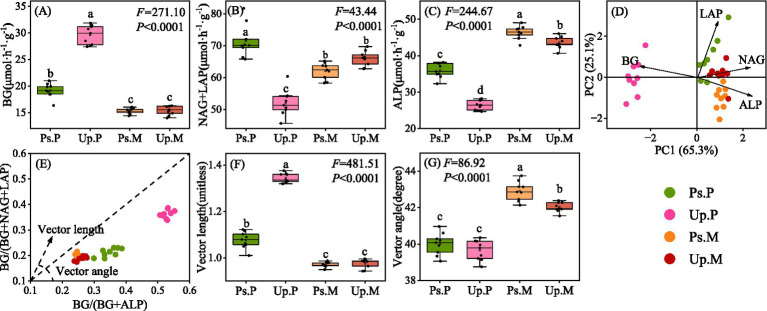
Effects of different dominant mycorrhizal types on soil enzyme activities and microbial resource limitations. **(A)** C-acquiring enzyme: *β*-1,4-glucosidase (BG); **(B)** N-acquiring enzymes: *β*-1,4-*N*-acetylglucosaminidase (NAG), leucine aminopeptidase (LAP); **(C)** P-acquiring enzyme: alkaline phosphatase (ALP); **(D)** Principal component analysis of soil extracellular enzyme activities; **(E)** Enzymatic stoichiometry of the relative proportions of C to N acquisition versus C to P acquisition; **(F)** Vector length; and **(G)** Vector angle. *Ps.P*, *Pinus sylvestris* pure stand; *Up.P*, *Ulmus pumila* pure stand; *Ps.M*, *Pinus sylvestris* in mixed stand; *Up.M*, *Ulmus pumila* in mixed stand. Significant differences (*p* < 0.05) between treatments are indicated by different lowercase letters.

The activity of C-acquiring enzymes was highest in Up.P (29.60 μmol h^−1^), which was 1.94 and 1.92 times that of Ps.M and Up.M, respectively (*p* < 0.05, Figure 2A). N-acquiring enzymes reached the highest activity in Ps.P (71.49 μmol h^−1^) (*p* < 0.05, Figure 2B). The activity of P-acquiring enzymes was elevated in Ps.M and Up.M soils at 43.37 and 43.57 μmol h^−1^, respectively (*p* < 0.05, Figure 2C). Mixed AM and EcM forests enhance the utilization efficiency of C resources through complementary C demands but also intensify microbial competition for P. Principal component analysis of extracellular enzyme activities revealed the highest microbial C limitation in Up.P (Figure 2D). The distribution of data points further indicated that Up.P experienced stronger microbial C and N limitations than Ps.P, whereas microbial resource limitations were alleviated in AM–EcM mixed stands. Vector length and vector angle, calculated from the relative proportions of extracellular enzyme activities, quantified microbial C and N limitations. All treatments had vector angles below 45°, suggesting that microbes were primarily limited by N rather than P. Compared with Up.P, vector length and angle in Up.M decreased from 1.34 to 0.98 and increased from 39.66° to 42.02°, respectively; compared with Ps.P, vector length and angle in Ps.M decreased from 1.08 to 0.97 and increased from 40.00° to 42.91°, respectively, with vector angles shifting toward 45° (Figures 2E–G). Compared with the pure stands, the vector angles in the mixed stands shifted toward 45°, accompanied by reduced vector lengths. This pattern indicates that microbial N limitation was alleviated, the N: P supply–demand balance became more equilibrated, and microbial C limitation was also weakened under mixed stands. It is important to note that all vector angles remained <45°, demonstrating that the mixed stands did not shift to P limitation. Instead, they transitioned from a strongly N-limited state toward a more balanced N–P stoichiometric condition.

### 3.3 Effects of tree mycorrhizal dominance on microbial biomass and community composition

**Figure 3:**
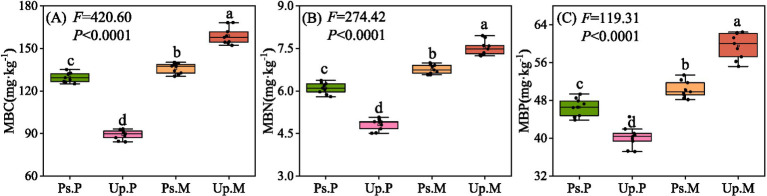
Soil microbial biomass in different dominant mycorrhizal forest types. **(A)** Microbial biomass C (MBC). **(B)** Microbial biomass N (MBN). **(C)** Microbial biomass P (MBP). Significant differences (*p* < 0.05) between treatments are indicated by different lowercase letters.

**Figure 4:**
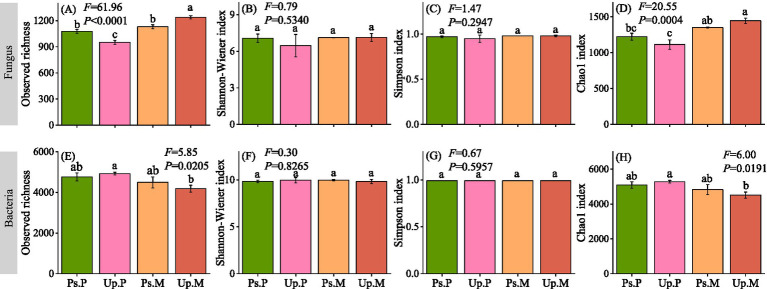
Fungal and bacterial community diversity indices in soils of different dominant mycorrhizal forest types. **(A)** Fungus observed richness; **(B)** fungus Shannon–Wiener index; **(C)** fungus Simpson index; **(D)** fungus Chao1 index; **(E)** bacteria observed richness; **(F)** bacteria Shannon–Wiener index; **(G)** bacteria Simpson index; and **(H)** bacteria Chao1 index. Significant differences (*p* < 0.05) between treatments are indicated by different lowercase letters.

**Figure 5:**
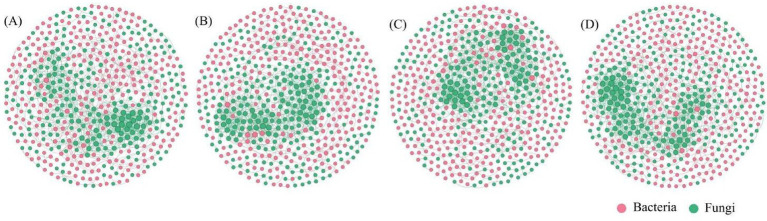
Microbial co-occurrence networks in the rhizosphere soils of forests dominated by different mycorrhizal types. The size of the dot represents the size of the microbial group. **(A)** *Pinus sylvestris* pure stand (Ps.P); **(B)** *Ulmus pumila* pure stand (Up.P); **(C)** *Pinus sylvestris* in mixed stand (Ps.M); and **(D)** *Ulmus pumila* in mixed stand (Up.M).

MBC, MBN, and MBP reached the highest values in Up.M, with 158.91, 7.52, and 59.53 mg g^−1^, respectively, which were significantly higher than in other stand types (*p* < 0.05, Figure 3). By contrast, the lowest values of MBC, MBN, and MBP were observed in Up.P (89.13, 4.81, and 40.25 mg g^−1^, respectively). Mixed AM–EcM stands significantly enhanced microbial biomass in the rhizosphere soils compared with AM or EcM monocultures (*p* < 0.05). Fungal community diversity was higher in Ps.P than in Up.P but lower than in Ps.M and Up.M (*p* < 0.05, Figures 4A–D). Conversely, bacterial community diversity was highest in Up.P compared with Ps.P, Ps.M, and Up.M (*p* < 0.05, Figures 4E–H). Network analysis revealed that the rhizosphere microbial network of Up.P had the lowest values in terms of node number, edge number, average degree, average path length, and average clustering coefficient (528, 1,002, 3.79, 8.82, and 0.38, respectively), compared with Ps.P (530, 1,012, 3.82, 8.94, and 0.42, respectively) (Figure 5, Table 2). Among the four stand types, Up.M showed the most complex rhizosphere microbial network, with the highest node number, edge number, average degree, and clustering coefficient (542, 1,164, 4.30, and 0.43, respectively), followed by Ps.M (530, 1,025, 3.86, and 0.43), and it exhibited the highest modularity (0.73). These results suggest that the combination of AM and EcM tree species enhances the complexity and stability of co-occurrence networks within rhizosphere microbial communities.

**Table 2:** Topological parameters of microbial co-occurrence networks in forests dominated by different mycorrhizal types.

Treatment	Node number	Edge number	Average degree	Average path length	Average clustering coefficient	Modularity
Ps.P	530	1012	3.82	8.94	0.42	0.71
Up.p	528	1002	3.79	8.82	0.38	0.72
Ps.M	530	1025	3.86	8.83	0.43	0.73
Up.M	542	1164	4.30	8.64	0.43	0.70

### 3.4 Relationships among soil chemical properties, microbial biomass, and microbial resource limitation

**Figure 6:**
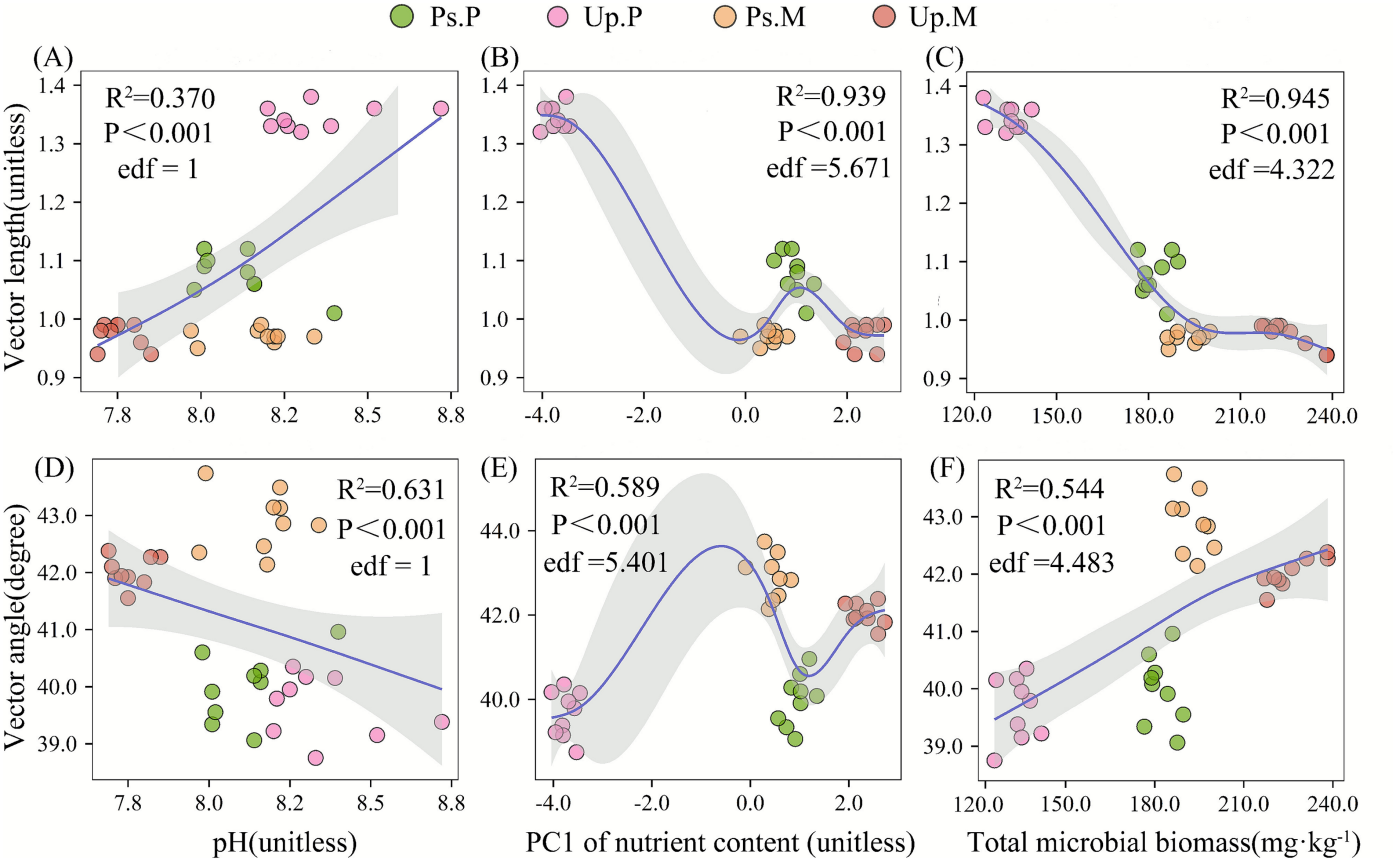
Microbial nutrient limitation in relation to soil resources under different mycorrhizal dominance: **(A)** Vector length: soil pH; **(B)** vector length: PC1 of soil nutrient content; **(C)** vector length: total microbial biomass; **(D)** vector angle: soil pH; **(E)** vector angle: PC1 of soil nutrient content; and **(F)** vector angle: total microbial biomass.

The GAM results showed that pH exerted a relatively weak effect on vector length and displayed an approximately linear increasing trend (Figure 6A). In contrast, soil nutrient content PC1 and microbial biomass had the highest explanatory power for vector length (*R*^2^ = 0.939 and 0.945, respectively) and exhibited strongly nonlinear responses (edf = 5.671 and 4.322, Figures 6B,C), indicating that they are the primary drivers of microbial C limitation. Changes in vector angle were mainly controlled by pH (*R*^2^ = 0.631, edf = 1D), whose influence was stronger than that of soil nutrient content PC1 (*R*^2^ = 0.589, Figure 6E) and microbial biomass (*R*^2^ = 0.544, Figure 6F).

**Figure 7:**
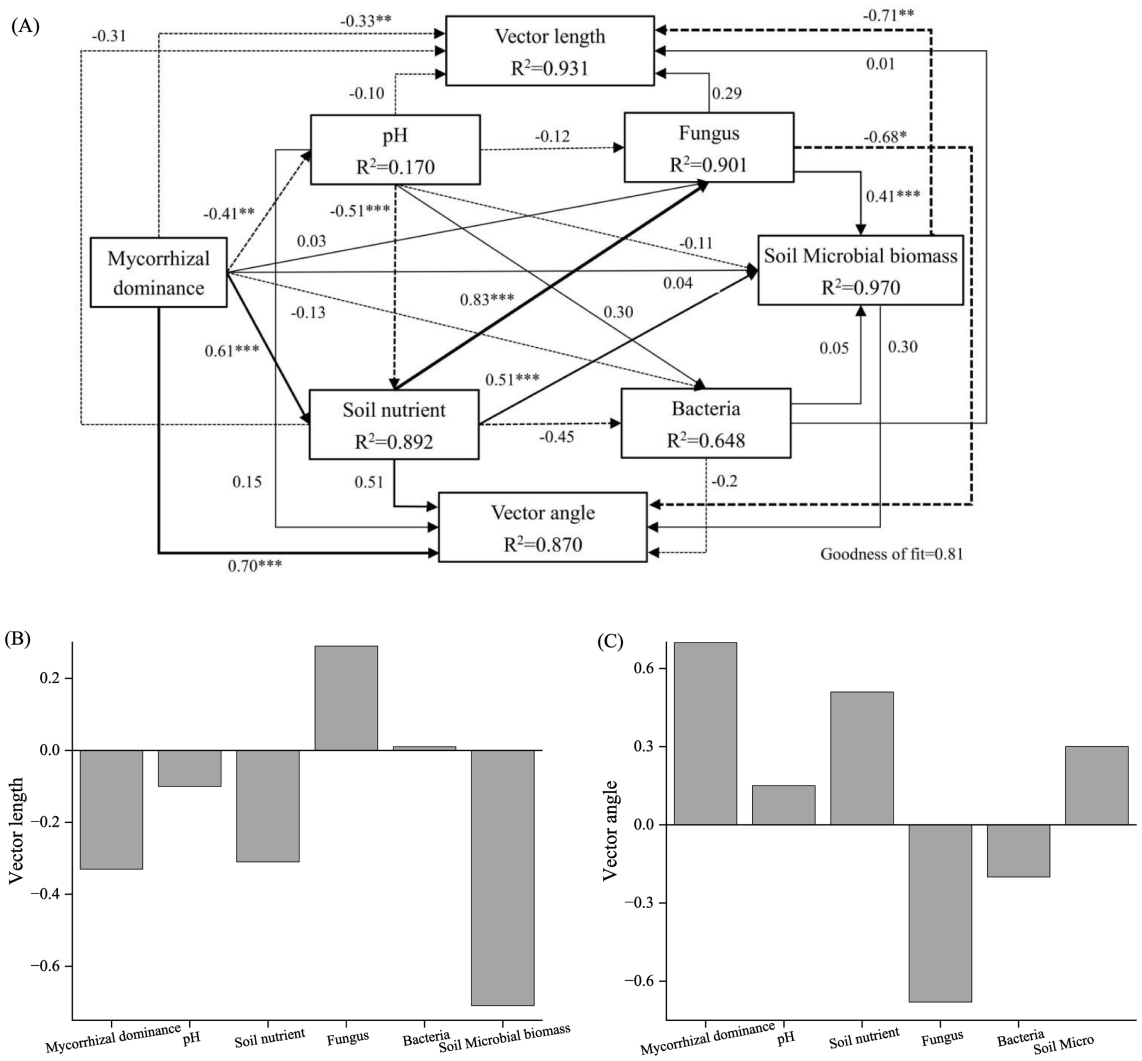
Partial least squares path model (PLS-PM) illustrating the direct and indirect effects of tree mycorrhizal dominance, soil pH, soil nutrients, fungal diversity, bacterial diversity, and soil microbial biomass on vector length (microbial C limitation) and vector angle (microbial N limitation). Solid and dashed arrows represent positive and negative effects, respectively. The significance of standardized path coefficients is indicated by numbers on the arrows. (*, **, and *** denote significant differences at the *p* < 0.05, *p* < 0.01, and *p* < 0.001 levels, respectively). *R*^2^ represents the explained variance of individual variables. Mycorrhizal dominance is treated as categorical, representing stand types (i.e., *Pinus sylvestris* var. *mongolica* monoculture, *Ulmus pumila* monoculture, and *Pinus × Ulmus* mixed stands); soil nutrient (i.e., total carbon, total nitrogen, total phosphorus, organic carbon, available nitrogen, and available phosphorus); fungus (i.e., fungus observed richness and fungus Chao1 index); bacteria (i.e., bacteria observed richness and bacteria Chao1 index); and soil microbial biomass (i.e., soil microbial biomass carbon, soil microbial biomass nitrogen, and soil microbial biomass phosphorus). Figure 7 presents direct path coefficients only; total effects reported in the main text represent the sum of direct and indirect effects.

We further applied PLS-PM to evaluate the effects of dominant mycorrhizal type, soil nutrients, pH, fungal diversity, bacterial diversity, and microbial biomass on vector length (microbial C limitation) and vector angle (microbial N limitation), with the model achieving a goodness of fit index of 0.81. The model demonstrated that stand configuration significantly influenced soil nutrients, pH, and microbial processes through direct and indirect pathways, explaining 81% of microbial nutrient limitation (Figure 7A). The direct effects indicated that tree mycorrhizal dominance was a crucial factor of microbial nutrient limitation. Specifically, mycorrhizal dominance significantly decreased soil pH and vector length (path coefficients = −0.41 and −0.33, *p* < 0.01) but strongly increased soil nutrient content and vector angle (path coefficients = 0.61 and 0.70, *p* < 0.001). The total effects of PLS-PM further revealed that pH exerted an overall positive effect on vector length (path coefficient = 0.31) and a negative effect on vector angle (path coefficient = −0.02) by influencing nutrient content (path coefficient = −0.51, *p* < 0.001) and microbial biomass (path coefficient = −0.11). Soil nutrients and microbial biomass showed direct negative effects on vector length but positive effects on vector angle, with microbial biomass significantly reducing vector length (path coefficient = −0.77, *p* < 0.01, Figure 7B, C). These findings suggest that mixing AM- and EcM-associated tree species can effectively improve soil nutrient status and alleviate microbial carbon and nitrogen limitations. For clarity, Figure 7 displays only the direct path coefficients, whereas the total effects reported in the text represent the overall effects (i.e., the sum of direct and indirect influences).

## 4 Discussion

### 4.1 Effects of AM and EcM monocultures on rhizosphere soil

The competition for nutrients between different mycorrhizal types regulates soil nutrient cycling (Seidel et al., 2024). EcM ecosystems typically rely on the mineralization of organic nutrients and fungus-mediated decomposition processes. This nutrient acquisition strategy satisfies plant nitrogen and phosphorus demands and inhibits the mineralization of organic carbon, thereby promoting SOC accumulation and maintaining elevated nutrient availability (Averill et al., 2016; Tunlid et al., 2022; Guan et al., 2025). By contrast, AM ecosystems primarily depend on the direct absorption of inorganic nutrients, exhibiting an increased demand for labile N and P. Consequently, soil nutrient dynamics in AM systems are more susceptible to environmental fluctuations than those in EcM systems (Faghihinia et al., 2024). Consistent with these mechanisms, our results showed that soils in AM monocultures had lower nutrient contents but significantly higher pH than those in EcM monocultures (*p* < 0.05). In EcM-dominated ecosystems, the competition for organic N and suppression of saprotrophic decomposers (the “Gadgil effect”) impede organic matter decomposition, enhancing the retention of soil C and organic nutrient pools (Gadgil and Gadgil, 1971; Orwin et al., 2011). Similarly, Averill et al. (2014) demonstrated that EcM-dominated ecosystems store more SOC than AM-dominated ones. The study area is located in a typical arid-to-semiarid ecological zone. The soil, which is alkaline and has low humidity, tends to maintain AM fungal communities that are resistant to high pH and have rapid metabolism. However, it is also more susceptible to microbial limitations, thereby restricting the effective supply capacity of soil nutrients (Barea et al., 2011). Enzyme activities and microbial community structures further revealed these contrasting mechanisms. Brzostek et al. (2015) showed that EcM fungi preferentially produce key enzymes involved in the degradation of complex organic matter, such as NAG, LAP, and ALP, to acquire organic nutrients. Similarly, our study found that NAG, LAP, and ALP activities; microbial biomass C, N, and P; and fungal diversity were significantly higher in EcM monocultures than in AM monocultures (*p* < 0.05). Conversely, AM monocultures exhibited higher BG activity, stronger microbial C limitation (vector length), N limitation (vector angle) (*p* < 0.05), and higher bacterial diversity than EcM monocultures. This pattern may stem from the higher pH and lower soil moisture observed in AM pure forests. In such alkaline and low-moisture environments, bacterial taxa with greater osmotic-stress tolerance and rapid metabolic capacity are more likely to be selected and maintain higher diversity. Consequently, bacterial diversity in AM pure forests is higher than that in EcM pure forests. Zheng et al. (2022) reported that AM tree species in European temperate forests are associated with increased microbial activity and bacterial diversity. The discrepancy in our results may be due to differences in climate, soil pH, and site conditions. The study area is located in a typical arid–semiarid ecosystem, characterized by generally low soil moisture, limited total inorganic nitrogen, and a relatively high soil C: N stoichiometry. These abiotic constraints inherently restrict microbial decomposition and nutrient regeneration rates, thereby exacerbating microbial demands for C and N and resulting in stronger microbial nutrient limitations. In contrast, the study site investigated by Zheng et al. is situated in a temperate European forest with higher soil fertility and a more neutral pH, where AM-associated tree species can sustain greater microbial biomass under more nutrient-rich conditions. Therefore, differences in microbial activity and nutrient limitation observed across regions—or even between forest types dominated by AM versus EcM trees—may partly arise from environmental filtering effects imposed by local abiotic stressors on extracellular enzyme investment patterns and microbial metabolic efficiency. This mechanism indicates that environmental constraints and mycorrhizal type jointly shape microbial nutrient acquisition strategies and patterns of resource limitation. Microbial network analysis further revealed that EcM monocultures had higher node number, edge number, average degree, average path length, clustering coefficient, and modularity than AM monocultures, indicating complex and highly connected microbial communities. This may be attributed to the extensive EcM hyphal networks that promote the decomposition of complex organic matter and nutrient release, thereby enhancing microbial biomass and network stability. By contrast, the simplified microbial networks in AM systems are likely driven by high soil pH and nutrient scarcity. Overall, the contrasting patterns observed in AM and EcM rhizospheres not only reflect the fundamental differences in their nutrient acquisition strategies but also highlight their far-reaching impacts on soil C, N, and P cycling, as well as on the stability of microbial interaction networks.

### 4.2 Effects of AM–EcM mixed stands on rhizosphere soil properties

Compared with monocultures, mixed forests usually exhibit enhanced environmental heterogeneity, which influences soil nutrient cycling and microbial processes at both the rhizosphere and stand scales (Kang et al., 2022; Jiang et al., 2017). The coexistence of multiple tree species in mixed stands creates diverse microhabitats and resource niches, thereby supporting a broad range of soil microorganisms, particularly fungi (Gould et al., 2016). This increased diversity often results in higher structural and functional complexity, yielding more stable and complementary ecological niches. At the stand scale, mixed forests exhibit greater litter diversity, with pronounced differences in stoichiometric traits, lignin content, and decomposition rates. Such heterogeneity in litter inputs can alter the composition of soil organic substrates, enhance microbial decomposition potential, and ultimately increase nutrient regeneration efficiency at the ecosystem level (Hättenschwiler et al., 2005; Prescott and Grayston, 2013). At the rhizosphere scale, AM and EcM trees display markedly complementary nutrient acquisition strategies: EcM fungi degrade organic substrates and promote the mineralization of nitrogen and phosphorus, whereas AM fungi efficiently utilize inorganic N and P. This complementary mechanism diversifies the nutrient sources available in mixed forests and enhances soil organic matter accumulation. Previous studies have shown that the coexistence of AM and EcM tree species can enhance soil resource-use efficiency (Deng et al., 2023; Singavarapu et al., 2022). Our study found that, except for SOC, the nutrient contents in the rhizosphere soil of *P. sylvestris* var. *mongolica* in mixed stands were lower than those in its pure stands (*P* < 0.05), while soil pH was higher than in the pure stands. This pattern may be attributed to enhanced nutrient transfer into plant and microbial biomass, as well as the release of alkaline cations such as Ca and Mg during litter decomposition, which can increase soil pH (Vesterdal et al., 2013). In mixed forests, the nutrient contents in the rhizosphere soil of *U. pumila* were all significantly higher than those in its pure stands, whereas soil pH was significantly reduced. This may result from AM tree species utilizing EcM-mediated organic nutrient mineralization, thereby enhancing P and N uptake efficiency. In addition, AM trees can lower soil pH through increased cation uptake, proton release, and organic acid secretion (Clarholm et al., 2015). The complementary stoichiometric traits of mixed litter and differential decomposition processes may accelerate SOC accumulation and nutrient turnover, particularly in AM-dominated ecosystems where nutrient limitation is pronounced. Luo et al. (2023) also demonstrated that, although fungal communities dominated by extracellular enzyme-producing groups have been shown to decompose organic matter slowly in any soil environment, the presence of herbaceous AM plants in the understory of such enzyme-dominated forests can accelerate the decomposition of soil organic matter. If AM tree species similarly promote decomposition, nutrient cycling in mixed forests may exceed that in stands dominated by a single mycorrhizal type. Furthermore, functional differences between AM and ECM may lead to complementary effects, enhancing productivity and alleviating microbial resource limitations in forests that adopt a mixed mycorrhizal strategy. Our study showed that vector length in mixed stands significantly decreased but vector angle significantly increased than in monocultures (*p* < 0.05). This indicates that mixed mycorrhizal strategies not only accelerate nutrient cycling and optimize the stoichiometric structure of organic matter decomposition and nutrient release but also move microbial nutrient acquisition close to the theoretical balance of C: N: P (1:1:1). As a result, microbial nutrient limitations are alleviated, promoting coordinated and balanced microbial resource use (Cheeke et al., 2017; Wu et al., 2023). This pattern may stem from the complementary functions of AM and EcM fungi jointly regulating organic matter decomposition and nutrient regeneration. EcM fungi enhance the mineralization of organic nitrogen and phosphorus, whereas AM fungi efficiently absorb inorganic P, thereby increasing the overall availability of soil N and P. At the same time, mixed forests increase SOC and microbial biomass, improving the structure of substrate supply and allowing microbes to acquire C, N, and P in a more balanced manner rather than investing disproportionately in a single class of extracellular enzymes. Previous studies have also noted that mixed-mycorrhizal systems can enhance enzymatic diversity and complementarity, enabling microbes to maintain stoichiometric balance, minimize metabolic costs, and improve resource-use efficiency (Camenzind et al., 2018; Moorhead and Sinsabaugh, 2006). Several studies (Banerjee et al., 2018; Wagg et al., 2019) have reported that mixed mycorrhizal strategies can increase fungal diversity and microbial biomass and enhance interactions and metabolic complementarity among functional groups, thereby forming highly interconnected microbial networks. Consistent with this, we found that MBC, MBN, MBP, and fungal diversity were significantly higher in mixed stands than in monocultures (*p* < 0.05), and microbial networks exhibited high complexity. By contrast, bacterial diversity was slightly reduced (*p* > 0.05), possibly due to shifts in organic matter inputs and strengthened fungal functions, which may partly replace bacterial ecological niches (Fierer et al., 2009).

### 4.3 Relationships among microbial nutrient limitation, mycorrhizal dominance, soil chemistry properties, and microbial biomass

Mixed forests, through a mixed-mycorrhizal strategy, promote complementary nutrient use between AM and EcM trees, thereby improving soil nutrient supply and microbial resource acquisition (Guo and Gong, 2024; Chen et al., 2020). By integrating the efficient inorganic nutrient uptake of AM fungi with the organic matter decomposition capacity of EcM fungi, mixed forests optimize soil nutrient availability and restructure microbial communities. This restructuring enhances fungal diversity and microbial network complexity, which in turn alleviates microbial C and N limitations. For example, Camenzind et al. (2018) found that mixed forests in the tropics increase microbial biomass and alter extracellular enzyme allocation patterns, thereby reducing microbial nutrient limitation. Our PLS-PM results further revealed that mycorrhizal dominance exerts a significant direct regulatory effect on microbial nutrient limitation. Mycorrhizal dominance had a highly significant positive direct effect on vector angle (path coefficient = 0.70, *p* < 0.001), suggesting that the dominant mycorrhizal type can directly alter the balance of microbial investment in N-acquiring extracellular enzymes. Microbial biomass and mycorrhizal dominance exerted strong negative effects on vector length (path coefficients = −0.71 and −0.33, respectively, *p* < 0.01), indicating that the dominant mycorrhizal type can directly modify the balance of microbial investment in N-acquiring extracellular enzymes. These results suggest that changes in microbial biomass and mycorrhizal dominance alter the distribution of extracellular enzymes among C, N, and P, serving as the principal factors in alleviating microbial resource limitations. Mycorrhizal dominance not only exerts direct effects but also indirectly regulates microbial resource balance by altering soil nutrient status and microbial biomass. The dominance of mycorrhizae exerted a significant negative effect on soil pH (path coefficient = −0.41, *p* < 0.01). The total effects of PLS-PM revealed that pH exerted an overall positive effect on vector length (path coefficient = 0.31) and a negative effect on vector angle (path coefficient = −0.02) by influencing nutrient content (path coefficient = −0.51, *p* < 0.001) and microbial biomass (path coefficient = −0.11). This result was aligned with the findings reported by Yuan et al. (2023), who found that mixed forests enhance nutrient availability by regulating litter decomposition and rhizosphere processes that modify soil acidity. In addition, mycorrhizal dominance had a strong positive effect on soil nutrients (0.61, *p* < 0.001), and nutrient enrichment indirectly promoted fungal diversity (0.83, *p* < 0.001) and microbial biomass (0.51, *p* < 0.001). These findings indicate that nutrient availability directly enhances fungal involvement in organic matter decomposition and nutrient uptake, as well as overall microbial metabolic potential. Notably, fungal diversity had a significant negative effect on vector angle (−0.68, *p* < 0.05), suggesting that increased fungal proportions shift extracellular enzyme allocation toward P acquisition, thereby increasing N availability; this response was likely associated with the ability of EcM fungi to access organic P (Sannakajsa et al., 2014). Furthermore, microbial biomass exhibited a negative effect on vector length but a positive effect on vector angle. The GAM analysis further revealed the environmental response patterns underlying microbial resource limitation. The results showed that pH exhibited linear effects on both vector length and vector angle (edf = 1), indicating that soil pH acts as a stable environmental gradient that drives microbial extracellular enzyme investment strategies. Nutrient PC1 achieved the highest explanatory power for vector length and vector angle (*R*^2^ > 0.90), demonstrating that the supply structure of soil C, N, and P fundamentally determines the direction of microbial resource-limitation responses. Meanwhile, the negative effect of microbial biomass on vector length suggests that higher metabolic capacity can alleviate C limitation, although such alleviation relies on external resource supply rather than increased microbial demand. Notably, GAM captures the overall response along environmental gradients, emphasizing a “supply-driven” mechanism—meaning that nutrient supply structure sets the background pattern of microbial limitation. In contrast, PLS-PM, after controlling for environmental factors, reveals a “demand-driven” mechanism—where microbial biomass and mycorrhizal function actively mitigate resource limitation through adjustments in extracellular enzyme allocation. Therefore, the combined evidence from this study indicates that microbial nutrient limitation is shaped not by supply-side or demand-side factors alone, but by a dual regulatory mechanism involving both nutrient supply conditions and microbial metabolic demand. These findings highlight that mixed forests, compared with monocultures, alleviate microbial resource limitations through multiple regulatory pathways, emphasizing the critical role of mixed-mycorrhizal strategies in maintaining ecosystem function and nutrient cycling balance. It should be noted that this study is based on rhizosphere soil, whose microenvironment is strongly influenced by root exudates, mycorrhizal symbiosis, and the interaction between rhizosphere microorganisms. Therefore, the microbial resource limitation patterns and enzyme activity regulation mechanisms revealed in this study mainly reflect the characteristics of the rhizosphere. In large soil masses or deeper soil layers, due to the significantly weakened influence of roots, the microbial community structure, enzyme investment strategies, and their responses to C, N, and P limitations may present different patterns. Therefore, the explanations of the related mechanisms in this study should be limited to the rhizosphere scale.

## 5 Conclusion

Our study highlights the regulatory mechanisms governing soil microbial community structure and nutrient limitations under different tree mycorrhizal dominances. We found that AM pure forests, characterized by high BG activity, maintained higher bacterial diversity but were accompanied with intensified microbial C and N limitations. By contrast, EcM pure forests demonstrated elevated NAG + LAP and ALP activities. Mixed forests of *U. pumila* and *P. sylvestris* var. *mongolica* demonstrated a synergistic effect of mixed mycorrhizal associations, which significantly enhanced soil SOC and microbial biomass, regulated extracellular enzyme activities, alleviated microbial nutrient limitations, and promoted the formation of complex and stable microbial interaction networks. PLS-PM further revealed that mycorrhizal dominance and microbial biomass were the primary factors alleviating microbial nutrient limitations. Overall, tree mycorrhizal dominance influences microbial nutrient limitations through variations in extracellular enzyme activities and microbial community assembly. In particular, AM–EcM mixed forests can achieve resource complementarity via mixed mycorrhizal strategies, thereby alleviating microbial nutrient limitations and enhancing the stability of forest soil ecosystems. These findings provide important theoretical support for the restoration of degraded forests, the optimization of mixed-forest configurations, and ecological rehabilitation in sandy land regions.

## Data Availability

Raw data have been deposited to National Center for Biotechnology Information (NCBI) under the BioProject number PRJNA1379231.

## Footnotes

1
https://maarjam.ut.ee/
